# Development of a chemiluminescence assay for tissue plasminogen activator inhibitor complex and its applicability to gastric cancer

**DOI:** 10.1186/s12896-024-00850-9

**Published:** 2024-05-08

**Authors:** Yu Ji, Yan Qin, Qi Tan, Yanru Qiu, Shuang Han, Xiaowei Qi

**Affiliations:** 1 Department of Pathology, Affiliated Hospital of Jiangnan University, No.1000, Hefeng Road, Wuxi, 214122 Jiangsu China; 2 Wuxi Medical College, Jiangnan University, Wuxi, Jiangsu, China

**Keywords:** Tissue plasminogen activator inhibitor complex, Chemiluminescence kit development, Gastric cancer, Venous thromboembolism, Stage

## Abstract

**Background:**

Venous thromboembolism (VTE), is a noteworthy complication in individuals with gastric cancer, but the current diagnosis and treatment methods lack accuracy. In this study, we developed a t-PAIC chemiluminescence kit and employed chemiluminescence to detect the tissue plasminogen activator inhibitor complex (t-PAIC), thrombin-antithrombin III complex (TAT), plasmin-α2-plasmin inhibitor complex (PIC) and thrombomodulin (TM), combined with D-dimer and fibrin degradation products (FDP), to investigate their diagnostic potential for venous thrombosis in gastric cancer patients. The study assessed variations in six indicators among gastric cancer patients at different stages.

**Results:**

The t-PAIC reagent showed LOD is 1.2 ng/mL and a linear factor R greater than 0.99. The reagents demonstrated accurate results, with all accuracy deviations being within 5%. The intra-batch and inter-batch CVs for the t-PAIC reagent were both within 8%. The correlation coefficient R between this method and Sysmex was 0.979. Gastric cancer patients exhibited elevated levels of TAT, PIC, TM, D-D, FDP compared to the healthy population, while no significant difference was observed in t-PAIC. In the staging of gastric cancer, patients in III-IV stages exhibit higher levels of the six markers compared to those in I-II stages. The ROC curve indicates an enhancement in sensitivity and specificity of the combined diagnosis of four or six indicators.

**Conclusion:**

Our chemiluminescence assay performs comparably to Sysmex’s method and at a reduced cost. The use of multiple markers, including t-PAIC, TM, TAT, PIC, D-D, and FDP, is superior to the use of single markers for diagnosing VTE in patients with malignant tumors. Gastric cancer patients should be screened for the six markers to facilitate proactive prophylaxis, determine the most appropriate treatment timing, ameliorate their prognosis, decrease the occurrence of venous thrombosis and mortality, and extend their survival.

**Supplementary Information:**

The online version contains supplementary material available at 10.1186/s12896-024-00850-9.

## Background

Gastric cancer is a disease of global significance. It is estimated that more than one million new cases are diagnosed each year, making it the fifth most common malignancy worldwide [1]. The average survival rate in advanced stages is less than 12 months [2]. Approximately 990,000 people worldwide are diagnosed with gastric cancer each year, of which approximately 738,000 dies [3]. Venous thromboembolism (VTE) is one of the major complications and a leading cause of death in patients with malignant tumors [4, 5]. VTE includes deep vein thrombosis (DVT) and pulmonary embolism (PE). Its insidious onset not only causes swelling and pain in the affected limb, affecting the patient's mobility, but also threatens the patient's life by causing pulmonary embolism and even death in severe cases of thrombus dislodgement. A large epidemiologic study reported that approximately 20% of new cases of VTE are associated with underlying tumors [6]. The risk of VTE in cancer patients is significantly higher than in the general population, and cancer patients have an increased risk of developing VTE compared to non-cancer patients [7]. Patients with metastases have a 4-13 times higher risk of developing VTE [8, 9]. Furthermore, patients who have received treatment for tumors and are hospitalized have a higher likelihood of developing VTE [10]. VTE can significantly impact cancer patient survival rates and mortality [11]. Currently, diagnosing VTE through imaging remains the gold standard. Passive detection of D-Dimer and fibrin degradation products (FDP) after thrombosis is ineffective for early VTE diagnosis. For patients with malignant tumors and VTE, the primary course of action is a 3-6 months treatment of the anticoagulant drug low molecular heparin [12]. This treatment has been known to improve the survival rate of tumor patients. However, VTE remains the second leading cause of death in patients with malignant tumors [13]. Therefore, early diagnosis and prediction of venous thrombosis in cancer patients is of utmost importance for preventative measures in clinical settings.

Tissue plasminogen activator inhibitor complex (t-PAIC) reflects vascular endothelial damage and the progression of the fibrinolytic system. It is formed through the 1:1 binding of tissue-type plasminogen activator (t-PA) to plasminogen activator inhibitor-1 (PAI-1), which is mostly associated with fibrinolytic inhibition. Thrombomodulin (TM) is a transmembrane glycoprotein that captures thrombin and exerts anticoagulant and anti-inflammatory effects. It is released into the bloodstream upon damage to the vascular endothelium. Elevated levels of TM indicate vascular endothelium damage in the patient. Thrombin-antithrombin III complex (TAT) is a 1:1 proportion of thrombin and antithrombin, reflecting the quantity of thrombin produced or the level of coagulation system activation. A heightened TAT level indicates a hypercoagulable state and a significant likelihood of thrombosis. Plasmin-α2-plasmin inhibitor complex (PIC) is formed when fibrinolytic enzymes bind 1:1 to α2 plasmin inhibitor, indicating the level of fibrinolytic enzyme production or activation of the fibrinolytic system. A high level of PIC suggests fibrinolysis or hyperfibrinolysis in the patient.

Six markers, specifically t-PAIC, TM, TAT, PIC, D-D, and FDP, were utilized in this study to evaluate the diagnostic efficacy of VTE in gastric cancer patients and to compare the thrombosis markers among gastric cancer patients with different stages.

## Results

### Optimization of antibody working concentration

The checkerboard experiment was performed with biotin-labeled antibody and HRP-labeled antibody, and different antibody concentrations were selected for detection to determine the RLU of 6 standards, and the results are shown in Supplementary Table 1. When the Biotin-Ab concentration is 2 μg/mL and the HRP-Ab concentration is 0.24 μg/mL, the signal value of each calibrator is suitable and the ratio is close, and the lower antibody concentration is selected under the same conditions to reduce the cost, and the combination was selected as the working concentration of the antibody in this study.

### Optimal incubation time

**Fig. 1:**
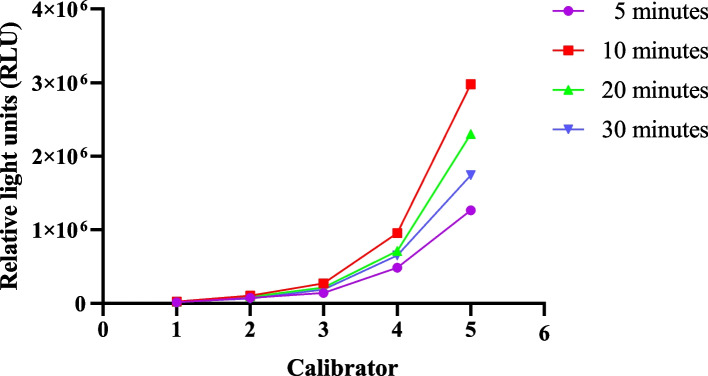
Influence of the 37 ℃ incubation time

The duration of the incubation period impacts RLU levels, and various time intervals were assessed to evaluate calibrators 1-5, plasma samples of high and low values. The outcomes are displayed in Fig. 1. An incubation period of 10 minutes yielded higher signal values across the board. Significantly reducing the testing time and improving detection throughput can be achieved by selecting a shorter incubation period. Consequently, this kit's reaction conditions were established at 37°C for 10 minutes.

### Evaluation of methodological performance

The method performance was evaluated according to the EP guidelines of the Clinical and Laboratory Standards Institute (CLSI).

#### Limit of detection

As demonstrated in Table 1, we calculated the mean and standard deviation of RLU for 20 standard dilutions. We then integrated M+2SD into the standard curve, resulting in concentration values of 1.104 ng/mL, 1.153 ng/mL, and 1.160 ng/mL. Consequently, we confirmed that the lowest detection limit for this method is 1.2 ng/mL.

**Table 1:** Verification results of the limit of detection

Indicators	Test1	Test2	Test3
M	39792	40308	40653
SD	2017	2244	2132
M+2SD	43825	44797	44916
LOD (ng/mL)	1.104	1.153	1.160

*Abbreviation*: *M* Mean, *SD* Standard deviation, *LOD* Limit of detection

#### Linearity

**Fig. 2:**
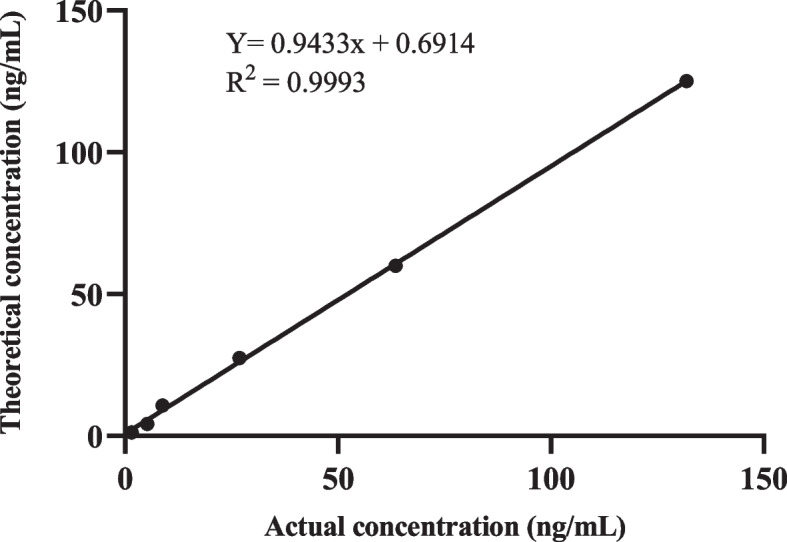
Linear testing of t-PAIC reagents

The high concentration samples were diluted using a gradient of low concentration samples to conduct the linearity test, as depicted in Fig. 2. The results demonstrate a strong, linear relationship between the theoretical and actual concentrations of the samples, as evidenced by the good linear regression equation obtained from fitting the data in Fig. 2: Y = 0.9433x + 0.6914, *R*>0.99.

#### Accuracy

As shown in Supplementary Table 2, the deviations of theoretical and measured concentrations were 4.62% and 0.98%, respectively, within ±10%.

#### Precision

Precision tests were conducted for both the low and high value quality controls (QCs), with Supplementary Table 3 documenting the results. The intra-batch coefficient of variation (CV) for the low-value QCs was 6.68%, 6.25%, and 5.66%, with an inter-batch CV of 6.74%. The high-value QCs had an intra-batch CV of 5.77%, 6.73%, and 5.64%, with an inter-batch CV of 6.03%. All the results were less than 10%.

#### Acceleration Stability

The experimental results showed that the deviation of the concentration of the QCs was less than 10% when the kit was placed at 37°C for different times compared to storage at 4°C (Table 2), indicating that the method demonstrates good stability.

**Table 2:** Verification results of thermal stability

Time	Samples	Labeled Concentrations	Measured Concentration			Bias		
			test1	test2	test3	test1	test2	test3
1 Day	Sample 1	4.83	4.79	5.07	4.51	-0.83%	4.97%	-6.63%
	Sample 2	16.62	17.17	16.92	17.52	3.31%	1.81%	5.42%
3 Day	Sample 1	4.83	5.12	4.87	4.67	6.00%	0.83%	-3.31%
	Sample 2	16.62	17.50	17.11	17.08	5.29%	2.95%	2.77%
6 Day	Sample 1	4.83	4.79	4.62	5.04	-0.83%	-4.35%	4.35%
	Sample 2	16.62	16.80	17.28	15.97	1.08%	3.97%	-3.91%
10 Day	Sample 1	4.83	4.58	5.02	4.63	-5.18%	3.93%	-4.14%
	Sample 2	16.62	15.30	17.01	15.30	-7.94%	2.35%	-7.94%

#### Clinical evaluation

**Fig. 3:**
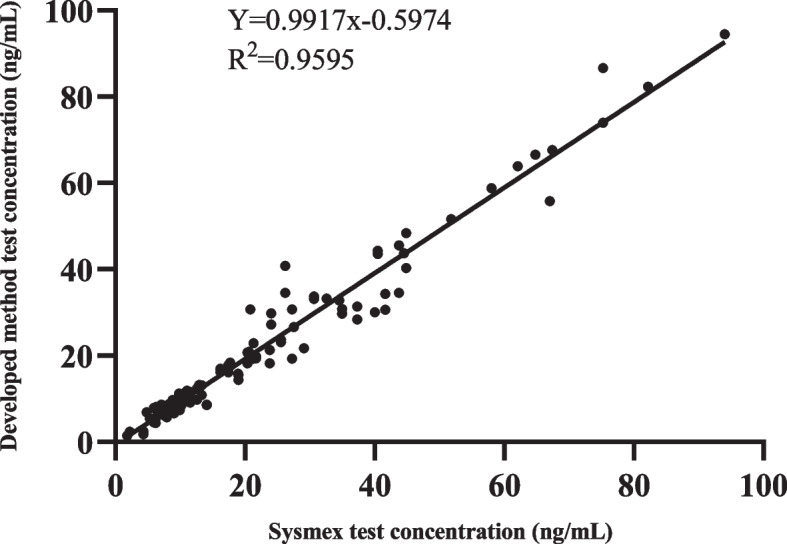
Comparison of the developed method to Sysmex

One hundred twenty copies of clinical plasma were tested by this method with the Japanese Sysmex kit. The correlation coefficient R was 0.979 was calculated after analysis, and there was good agreement between the two assays (Fig. 3).

### Comparison of t-PAIC, TAT, PIC, TM, D-D and FDP between control group and gastric cancer groups

As shown in Table 3, we found that TAT, TM, PIC, D-D, and FDP were significantly higher than those of the control group, and the differences were statistically significant (*P* < 0.001 or *P* < 0.05). t-PAIC did not show significant differences (*P* > 0.05).

**Table 3:** Comparison of each test index between gastric cancer population and healthy population

Groups	Control Group	GC Group	Z Value	P Value
N	80	293		
t-PAIC (ng/mL)	4.43 (2.96,6.60)	4.49 (3.00,6.89)	-0.288	0.773
TM (TU/mL)	6.75 (5.21,8.44)	7.84 (5.73,11.61)	-3.564	<0.001
TAT (ng/mL)	2.60 (2.13,3.71)	8.91 (5.75,12.89)	-12.171	<0.001
PIC (μg/mL)	0.32 (0.20,0.50)	0.68 (0.47,0.94)	-9.188	<0.001
D-D (mg/L)	0.36 (0.25,0.46)	0.85 (0.40,2.26)	-7.952	<0.001
FDP (mg/L)	3.35 (2.35,4.30)	3.40 (2.10,7.20)	-2.017	0.044

*Abbreviation*: *GC* Gastric cancer, *t-PAIC* Tissue plasminogen activator inhibitor complex, *TAT* Thrombin-antithrombin complex, *TM* Thrombomodulin, *PIC* Plasmin-α2-plamininhibitor complex, *D-D* D-Dimer, *FDP* Fibrinogen degradation product

* *P* < 0.05 considered statistically significant

### Comparison of t-PAIC, TAT, PIC, TM, D-D, and FDP in the thrombus and non-thrombus groups of gastric cancer patients

As shown in Table 4, t-PAIC, TAT, PIC, TM, D-D, and FDP were significantly higher in the thrombus group than in the non-thrombus group, and the differences were statistically significant (*P* < 0.001 or *P* < 0.05).

**Table 4:** Comparison of each test index in thrombosis group and non-thrombosis group of gastric cancer patients

Groups	Non-thrombosis group	Thrombosis group	Z Value	P Value
N	239	54		
t-PAIC (ng/mL)	4.43 (2.96,6.33)	5.24 (3.04,17.27)	-0.247	0.014
TM (TU/mL)	7.48 (5.62,10.74)	10.88 (6.85,13.87)	-3.505	<0.001
TAT (ng/mL)	7.67 (5.34,10.96)	16.99 (11.63,26.61)	-8.469	<0.001
PIC (μg/mL)	0.66 (0.45,0.90)	0.88 (0.53,1.48)	-3.113	0.002
D-D (mg/L)	0.75 (0.36,1.85)	1.78 (0.78,5.05)	-3.816	<0.001
FDP (mg/L)	3.20 (2.10,5.80)	6.15 (2.70,16.55)	-3.721	<0.001

*Abbreviation*: *GC* Gastric cancer, *t-PAIC* Tissue plasminogen activator inhibitor complex, *TAT* Thrombin-antithrombin complex, *TM* Thrombomodulin, *PIC* Plasmin-α2-plamininhibitor complex, *D-D* D-Dimer, *FDP* Fibrinogen degradation product

* *P* < 0.05 considered statistically significant

### Comparison of t-PAIC, TAT, PIC, TM, D-D, and FDP in gastric cancer stages I-II and III-IV

According to Table 5, t-PAIC, TAT, PIC, TM, D-D and FDP were significantly higher in stages III-IV than in stages I-II, and the difference was statistically significant (*P* < 0.001 or *P* < 0.05).

**Table 5:** Comparison of each test index in stages I-II and III-IV

Groups	Stage I-II	Stage III-IV	Z Value	*P* Value
N	112	181		
t-PAIC (ng/mL)	4.02 (2.81,5.72)	4.78 (3.20,7.40)	-2.603	0.009
TM (TU/mL)	7.26 (5.13,10.88)	8.26 (6.08,12.02)	-2.324	0.02
TAT (ng/mL)	6.69 (3.75,9.15)	10.36 (6.90,15.34)	-6.455	<0.001
PIC (μg/mL)	0.52 (0.37,0.68)	0.86 (0.60,1.08)	-7.502	<0.001
D-D (mg/L)	0.38 (0.22,0.69)	1.59 (0.75,3.36)	-9.874	<0.001
FDP (mg/L)	2.20 (1.70,3.20)	5.40 (3.00,11.00)	-9.164	<0.001

*Abbreviation*: *GC* Gastric cancer, *t-PAIC* Tissue plasminogen activator inhibitor complex, *TAT* Thrombin-antithrombin complex, *TM* Thrombomodulin, *PIC* Plasmin-α2-plamininhibitor complex, *D-D* D-Dimer, *FDP* Fibrinogen degradation product

* *P* < 0.05 considered statistically significant

### Evaluation of the value of each marker in the diagnosis of VTE by receiver operating characteristic (ROC) curve

**Fig. 4:**
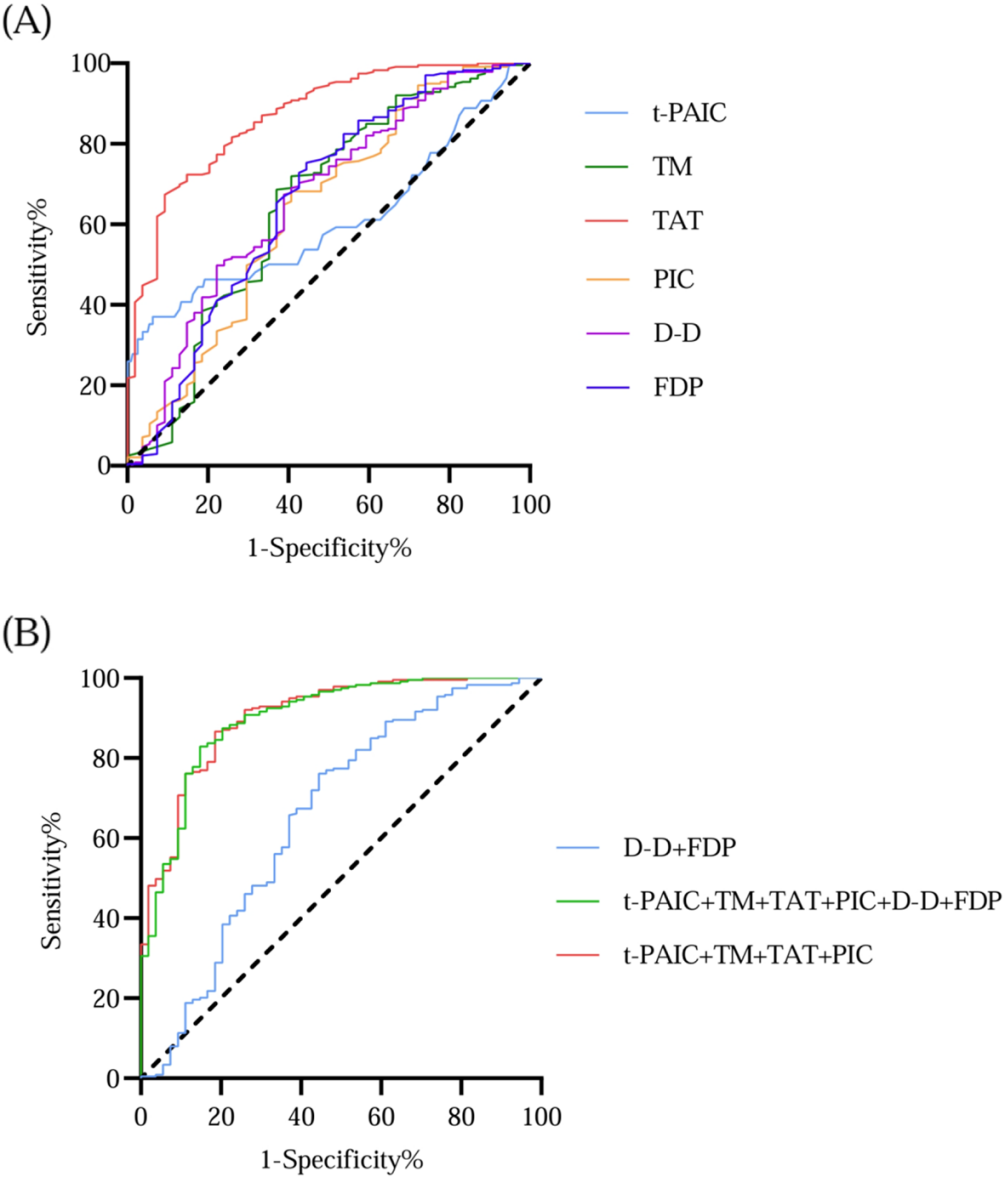
Diagnostic value of 6 markers for VTE. **A** Receiver operating characteristic (ROC) curve analysis of each marker for VTE. **B** Receiver operating characteristic (ROC) curve analysis of combined 6 markers in diagnosis of VTE

For the diagnosis of VTE, the area under the curve (AUC) of t-PAIC, TM, TAT, PIC, D-D, and FDP were 0.608, 0.653, 0.869, 0.636, 0.666, and 0.662, respectively. t-PAIC, TM, TAT, PIC, D-D, and FDP had critical values of 9.96 ng/ mL, 9.54 TU/mL, 9.61 ng/mL, 0.82 μg/mL, 1.40 mg/L, and 5.85 mg/L, respectively. TAT had a good diagnostic value for VTE, with a sensitivity of 90.7% and a specificity of 69%. t-PAIC had an AUC of 0.608, and it had the lowest diagnostic efficiency (Supplementary Table 4, Fig. 4). When the six markers were applied in combination, the AUC of "t-PAIC+TM+TAT+PIC" and "t-PAIC+TM+TAT+PIC+D-D+FDP" were significantly higher than that of "D-D+FDP" for VTE in gastric cancer patients. The AUC, sensitivity and specificity of "D-D+FDP" were 0.66, 55.6% and 76.2%, respectively, while the AUC, sensitivity and specificity of "t-PAIC+TM+TAT+PIC" were 0.90, 85.2% and 82.8%, respectively. The AUC, sensitivity and specificity of "t-PAIC+TM+TAT+PIC+D-D+FDP" were 0.904, 81.5% and 86.6%, respectively. (Supplementary Table 5, Fig. 4).

## Discussion

We have created a chemiluminescent immunoassay to detect t-PAIC in human plasma. Our t-PAIC kit exhibits excellent performance and overall stability while demonstrating a strong correlation with Sysmex methods, specifically. Clinical studies utilizing this method yielded reliable results and confirmed the suitability of the kit for clinical use. We evaluated the diagnostic value of each marker in venous thrombosis in gastric cancer patients by detecting the levels of t-PAIC, TM, TAT, and PIC in plasma using fully automated chemiluminescence. The method has benefits such as convenient sample collection, requiring a small sample volume, only 30 μL of venous plasma specimen for each test, and enabling large volume and rapid detection in the laboratory. The kits have a high sensitivity, and results are available in just 20 minutes. Additionally, they are cost-effective and perform similarly to Sysmex's methods.

The process of thrombosis is a complex series of complications associated with the coagulation, fibrinolytic, and endothelial systems [14, 15]. Currently, routine laboratory parameters for coagulation testing, such as D-dimer, FDP, prothrombin Time, activated partial thromboplastin time, thrombin time, fibrinogen and antithrombin III, etc. However, these parameters are passively detected after late thrombosis and are used to detect and screen for thrombosis in the later stage. Additionally, they are insensitive to the pre-thrombotic state and pre-diffuse intravascular coagulation (pre-DIC). Therefore, these tests may not effectively detect early signs of thrombosis. Thrombolytic therapy monitoring does not yield timely feedback, and there is still a dearth of reliable and early diagnosis.

TAT is a sensitive indicator of thrombin production and activation of the coagulation system [16, 17]. It is best to assess anticoagulant therapy when TAT is produced, as it can increase in the prethrombotic state. Therefore, the evaluation of TAT levels is ideal for the early diagnosis of thrombophilia and for monitoring thrombolytic therapy. A sustained increase in TAT indicates a higher risk of thrombosis. PIC serves as the starting point of the fibrinolytic system and indicates the extent of activation of fibrinolytic enzymes [18]. It also tracks the functional condition of the fibrinolytic system and guides antifibrinolytic therapy. TM functions as an indicator of the endothelial system, providing insight into the damage or recovery of blood vessel endothelium [19]. Damage to the vascular endothelium in patients with malignant tumors may elevate the level of TM [20]. Additionally, t-PAIC indicates not only abnormalities in the fibrinolytic system but also a correlation with endothelial damage [21]. Furthermore, TM and t-PAIC serve as efficient markers in predicting organ failure and clinical prognosis in patients with DIC and thrombosis [21–24].Compared to traditional coagulation processes, these four markers reflect the initial stages of blood coagulation within the body, detecting the emergence of blood clots in cancer patients with enhanced sensitivity and reliability. They also aid in the assessment of post-surgical thrombosis and bleeding, and determining the efficacy of thrombolysis and endothelial system impairment.

The study revealed a significant increase in TAT, TM, PIC, D-D, and FDP levels in gastric cancer patients when compared to healthy individuals. Moreover, the thrombus group exhibited a significantly higher elevation of t-PAIC, TAT, TM, PIC, D-D, and FDP in comparison to the non-thrombus group. ROC analysis results demonstrated that all six markers have a certain degree of diagnostic value for venous thrombosis in gastric cancer patients. TAT exhibited the highest diagnostic value, with an optimal cut-off value of 9.61 ng/mL nanograms per milliliter and an AUC of 0.869 demonstrate a superior diagnostic value over commonly used clinical indicators, such as D-D and FDP, for detecting venous thrombosis. While single marker application showed some value in the diagnosis of venous thrombosis, combining four or six indicators increased the AUC, sensitivity, and specificity. Specifically, the AUC, sensitivity and specificity for "t-PAIC+TM+TAT+PIC" were 0.90, 85.2% and 82.8%, respectively. The "t-PAIC+TM+TAT+PIC+D-D+FDP" produced an AUC of 0.904, with sensitivity and specificity resulting in 81.5% and 86.6%, respectively. We then proceeded to compare the four gastric cancer indexes across the different stages and found that as the stage progressed, the level of these indexes increased. Patients in stage III and IV had higher indicators compared to those in stage I and II.

The incidence of thrombosis is significantly affected by staging at malignancy diagnosis in all tumor types. For example, patients with uterine cancer and early staging had a lower incidence of thrombosis compared to patients with stage IV cancer [25, 26]. This finding is consistent with previous research that suggests cancer-associated thrombosis (CAT) might be a surrogate for aggressive tumor behavior in gynecologic malignancies [27]. It is noteworthy that cancer stage is not a component in several present CAT prediction models [28, 29], and the integration of disease ranges may enhance forthcoming predictive value.

Our study has some limitations. Specifically, the difference in t-PAIC levels between gastric cancer patients and healthy controls was not found to be significant. Our findings suggest that cancer cell bloodstream metastasis has a significant impact on local and systemic fibrinolysis, particularly in the development of tumor plugs in the abdominal wall. The release of TPA from tumor plugs apposed to the vessel wall leads to local fibrinolysis, which dissolves fibrin and causes tumor cells to appose to the vessel wall. This in turn facilitates further metastasis to other organs. Technical terms are clearly explained upon first use, and language throughout the text adheres to formal, objective principles without the use of biased, emotional, figurative, or ornamental language. Citations are consistent and adhere to style guide regulations. Therefore, t-PAIC levels were found to be elevated in patients with tumor bloodstream metastasis. The exclusion of gastric cancer patients who developed metastasis from this study may have impacted the absence of significant differences in t-PAIC levels between gastric cancer patients and healthy controls. Further investigation of t-PAIC's efficacy in gastric cancer patients who develop metastasis can be conducted in future research. Furthermore, the utilization of TM and t-PAIC have been linked to organ failure, warranting further investigation into their application in patients with different underlying diseases experiencing this issue.

VTE is the second leading cause of death among oncology patients. Tumor-related cases account for 20% to 30% of initial VTE diagnoses, with roughly 12% occurring in chemotherapy patients. The incidence of VTE among gastric cancer patients poses a significant challenge, impacting prognosis and survival rates. As such, it is imperative for gastrointestinal surgeons to address this complication. (1) To continue in-depth basic medical research to gain a deeper understanding of the pathogenesis of VTE in cancer patients; (2) To develop a standardized VTE prevention program for gastric cancer patients; (3) To continue the research and development of new medicines and improvement of existing medicines to reduce the probability of drug-induced adverse events; (4) Enhance the role of gastrointestinal surgeons in preventing and treating VTE in patients with gastric cancer; (5) Conduct further clinical studies to identify the factors that influence the incidence of VTE in gastric cancer patients, evaluate the effectiveness of preventive measures, and assess the incidence of adverse reactions.

## Conclusion

In summary, the current optimal non-invasive method for detecting VTE involves combining TAT, PIC, TM, t-PAIC, D-D, and FDP. This technique increases sensitivity and reliability in detecting thrombus formation and occurrence in gastric cancer patients, postoperative monitoring of thrombosis and bleeding, efficacy of thrombolytic therapy, and endothelial system impairment. It can screen early-stage gastric cancer patients at high risk for venous thrombosis, enabling targeted prevention and treatment. Moreover, it facilitates active patient use of preventive medication, identifies optimal treatment time, enhances patient prognosis, decreases venous thrombosis incidence and related fatalities, and extends patient survival time. It offers a new approach for preventing and managing venous thrombosis in patients with gastric cancer. This approach holds crucial theoretical and clinical significance.

## Material and Methods

### Reagents and materials

The t-PAIC antigen was purchased from Yarewell Biotechnology Ltd. (Shenzhen, China) and the t-PAIC antibody was provided by Baiming Biotechnology (Wuxi, China). Calibrators and substrates were provided by Baiming Biotechnology (Wuxi, China). The chemiluminescence and immunoluminescence instruments were purchased from Antu Experimental Instrument Company (Zhengzhou, China), and the pH meter was purchased from Mettler Toledo (Switzerland).

### Preparation of biotinylated antibody

Take 1 mg of encapsulated antibody, desalt the column replacement buffer for phosphate buffer and collect approximately 1 mL. Add 1 mg of activated biotin to 1 mL of phosphate buffer, take 100 µL to add to the antibody solution, mix well and allow to react for 1 hour at room temperature. The labelled Biotin-Ab was purified by addition to a desalting column. The absorbance of the Biotin-Ab was measured at 280 nm and the protein recovery was calculated as 94%.

### Preparation of horseradish peroxidase (HRP) binding antibody

HRP dissolved in deionized water was weighed and then mixed with NaIO4 (0.1 mol/L) solution. Finally, the reaction was dialyzed overnight in sodium acetate solution (1 mmol/L, pH 4.4) in a refrigerator at 4℃. The mixture was stirred for 20 minutes at room temperature while being protected from light. Afterward, the mixture was dialyzed in sodium acetate (1 mmol/L, pH 4.4) overnight in a refrigerator at 4℃. Next, 4 μL of ethylene glycol was added, and the reaction was performed for 30 minutes at room temperature while being protected from light. Finally, the reaction was dialyzed overnight in sodium acetate solution (1 mmol/L, pH 4.4) in a refrigerator at 4℃. Finally, the reaction was dialyzed overnight in sodium acetate solution (1 mmol/L, pH 4.4) in a refrigerator at 4°C. 4 microliters of ethylene glycol were added, and the reaction was carried out for 30 minutes at room temperature, protected from light. Next, add t-PAIC labeled antibody and dialyze it in carbonate buffer (0.2 mol/L, pH 9.5) at 4℃ overnight. After that, add 0.1 mL NaBH4 to the conjugate, mix it well, and react it at 4℃ for 2 hours under light protection. Then, add an equal volume of saturated ammonium sulfate under stirring and leave it at 4℃ for 1 hour. Finally, centrifuge it at 5000 r/min for 15 minutes and discard the supernatant. The solution dissolved the precipitate using PBS. Afterwards, glycerol was added in equal volume and stored at -20℃.

### Reaction process

**Fig. 5:**
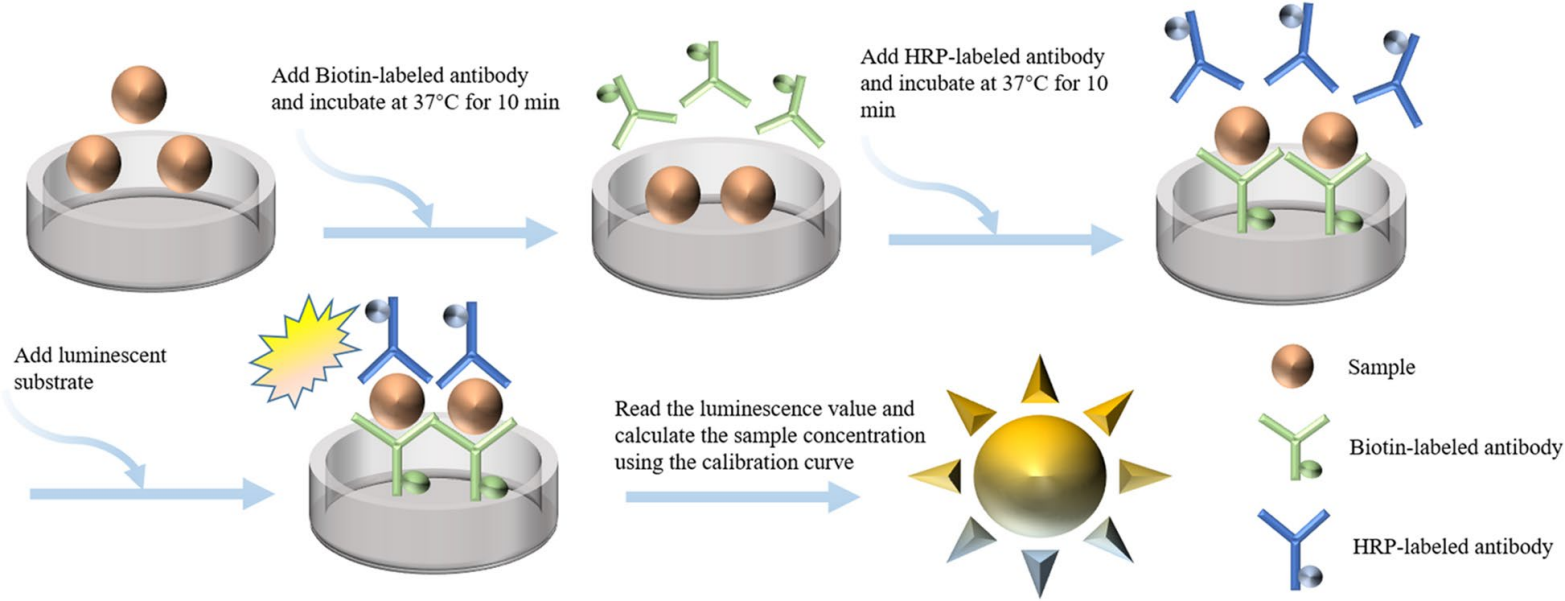
Reaction process

Thirty μL of sample and 80 μL of biotinylated antibody were added to a microtiter plate. The microtiter plate was incubated at 37℃ for 10 minutes and then washed 3 times with washing buffer. After patting dry, 80 μL of HRP-labeled antibody was added to the microtiter plate, which was again incubated at 37℃ for 10 minutes, then washed 3 times with washing buffer and patted dry. Finally, the substrate was added and relative light units (RLU) was measured. (Fig. 5)

### Study population

In this study, we included a total of 293 patients with gastric cancer in our case group. We excluded patients who had received coagulant drugs within 1 week prior to the study. Patients with gastric cancer confirmed by CT, MRI, histopathology, or cytology were included and their clinical stage was determined. The study comprised of 60 cases of stage I gastric cancer, 52 cases of stage II gastric cancer, 75 cases of stage III gastric cancer, and 106 cases of stage IV gastric cancer. Patients with deep vein thrombosis (DVT) confirmed by color Doppler ultrasound or angiography, or pulmonary embolism (PE) diagnosed by spiral CT pulmonary arteriography and MRI pulmonary arteriography were assigned to the thrombosis group. Meanwhile, 80 healthy individuals who received physical examinations in the hospital during the same period were selected as the control group. We excluded individuals with hyperlipidemia, diabetes mellitus, or coagulation-related diseases. This study was approved by the Institutional Ethics Committee.

### Data analysis

Data were analyzed and plotted using SPSS 26.0 and Graph Pad Prism 9.0. Normally distributed continuous variables were expressed as mean ± standard deviation and non-normally distributed as median [quartiles (P25-P75)]. The Mann-Whitney U test was used to compare differences between groups. Binary logistic regression was used to analyze the relationship between t-PAIC, TAT, PIC, TM, D-Dimer, FDP, and VTE. The diagnostic efficiency was evaluated using receiver operating characteristic (ROC) curve, and the maximum Youden index was used as the critical value. *P*<0.05 indicated a statistically significant difference.

### Supplementary Information

**Supplementary Material 1.** 

## Data Availability

The data used to support the findings of this study are available from the corresponding author upon request.

## Abbreviations

VTE: Venous thromboembolism
DVT: Deep vein thrombosis
PE: Pulmonary embolism
CAT: Cancer-associated thrombosis
t-PAIC: Tissue plasminogen activator inhibitor complex
TAT: Thrombin-antithrombin III complex
PIC: plasmin-α2-plasmin inhibitor complex
TM: Thrombomodulin
D-D: D-Dimer
FDP: Fibrin degradation products
RLU: Relative light units
CLSI: Clinical and Laboratory Standards Institute
M: Mean
SD: Standard deviation
LOD: Limit of detection
QCs: Quality controls
CV: Coefficient of variation
ROC: Receiver operating characteristic
AUC: Area under the curve
