# Supplementary material for: Development of a chemiluminescence assay for tissue plasminogen activator inhibitor complex and its applicability to gastric cancer
Source: BMC Biotechnol. 2024 May 8;24:30. doi: 10.1186/s12896-024-00850-9 (PMC11080135; doi:10.1186/s12896-024-00850-9)
Supplement: Supplementary file 1 — Supplementary Material 1. [file 12896_2024_850_MOESM1_ESM.docx]

**Supplementary Information**

Supplementary Table 1 t-PAIC Determining the concentration of Biotin-labeled antibody with t-PAIC HRP-labeled antibody

| t-PAIC Biotin-labeled antibody (μg/mL) | 2 | | | 2.6 | | | 3.2 | | |
| --- | --- | --- | --- | --- | --- | --- | --- | --- | --- |
| t-PAIC HRP-labeled antibody (μg/mL) | 0.12 | 0.24 | 0.36 | 0.12 | 0.24 | 0.36 | 0.12 | 0.24 | 0.36 |
| S1 | 11940 | 17058 | 20789 | 14621 | 24368 | 29042 | 22466 | 31678 | 44349 |
| S2 | 47667 | 68096 | 83773 | 58368 | 97280 | 119625 | 99164 | 126464 | 177049 |
| S3 | 148525 | 212179 | 204924 | 181868 | 303113 | 380015 | 320546 | 394047 | 551666 |
| S4 | 424040 | 605771 | 653297 | 519232 | 865387 | 960932 | 783369 | 1125003 | 1575004 |
| S5 | 1104125 | 1855673 | 1123547 | 1336084 | 2650961 | 2657927 | 1851064 | 2929312 | 3377324 |
| S2/S3 | 32% | 32% | 41% | 32% | 32% | 31% | 31% | 32% | 32% |
| S3/S4 | 35% | 35% | 31% | 35% | 35% | 40% | 41% | 35% | 35% |
| S4/S5 | 38% | 33% | 58% | 39% | 33% | 36% | 42% | 38% | 47% |

Supplementary Table 2 Verification results of accuracy

| Concentrations (ng/mL) | Test1 | Test2 | Test3 | Average  (g/mL) | Bias  (%) |
| --- | --- | --- | --- | --- | --- |
| 4.83 | 5.20 | 4.99 | 4.97 | 5.05 | 4.62% |
| 16.62 | 16.74 | 16.71 | 16.9 | 16.78 | 0.98% |

Supplementary Table 3 Verification results of precision

| Serial number | LOW | | | HIGH | | |
| --- | --- | --- | --- | --- | --- | --- |
|  | Batch1 | Batch2 | Batch3 | Batch1 | Batch2 | Batch3 |
| 1 | 4.52 | 4.97 | 4.93 | 17.28 | 15.79 | 17.45 |
| 2 | 4.83 | 5.26 | 4.97 | 16.95 | 17.25 | 16.12 |
| 3 | 5.17 | 4.64 | 4.88 | 15.46 | 15.96 | 16.29 |
| 4 | 4.64 | 5.22 | 5.07 | 17.68 | 17.78 | 14.96 |
| 5 | 4.49 | 4.69 | 5.02 | 15.62 | 15.14 | 15.79 |
| 6 | 4.59 | 4.83 | 4.42 | 15.55 | 15.79 | 15.62 |
| 7 | 5.37 | 5.31 | 4.44 | 15.15 | 18.45 | 15.79 |
| 8 | 5.12 | 5.68 | 4.73 | 15.98 | 15.79 | 14.21 |
| 9 | 4.44 | 4.97 | 4.35 | 15.14 | 17.18 | 16.62 |
| 10 | 5.02 | 5.17 | 4.64 | 16.79 | 15.53 | 16.45 |
| intra-batch M | 4.82 | 5.07 | 4.75 | 16.16 | 16.47 | 15.93 |
| intra-batch SD | 0.33 | 0.32 | 0.27 | 0.93 | 1.11 | 0.90 |
| intra-batch CV | 6.86% | 6.25% | 5.66% | 5.77% | 6.73% | 5.64% |
| inter-batch M | 4.88 | | | 16.19 | | |
| inter-batch SD | 0.33 | | | 0.98 | | |
| inter-batch CV | 6.74% | | | 6.03% | | |

Supplementary Table 4 Receiver operating characteristic (ROC) curve analysis for each detected marker

| Markers | Cut-off values | AUC (95%CI) | Sensitivity (%) | Specificity (%) | Youden Index |
| --- | --- | --- | --- | --- | --- |
| t-PAIC | 9.96 ng/mL | 0.608 (0.509-0.706) | 37.0 | 93.7 | 0.307 |
| TM | 9.54 TU/mL | 0.653 (0.562-0.743) | 63.0 | 68.6 | 0.316 |
| TAT | 9.61 ng/mL | 0.869 (0.818-0.920) | 90.7 | 69.0 | 0.597 |
| PIC | 0.82 μg/mL | 0.636 (0.546-0.725) | 59.3 | 68.2 | 0.275 |
| D-D | 1.40 mg/L | 0.666 (0.582-0.750) | 61.1 | 67.4 | 0.285 |
| FDP | 5.85mg/L | 0.662 (0.572-0.752) | 55.6 | 75.3 | 0.309 |

Abbreviation: GC: Gastric cancer; t-PAIC: Tissue plasminogen activator inhibitor complex; TAT: Thrombin-antithrombin complex; TM: Thrombomodulin; PIC: plasmin-α2-plamininhibitor complex; D-D: D-Dimer; FDP: Fibrinogen degradation product. * P < 0.05 considered statistically significant.

Supplementary Table 5 Analysis of Receiver operating characteristic (ROC) curve with combined 6 markers for diagnosis of VTE

| Markers | AUC (95%CI) | Sensitivity (%) | Specificity (%) | Youden  Index |
| --- | --- | --- | --- | --- |
| t-PAIC+TM+TAT+PIC | 0.900 (0.852-0.947) | 85.2 | 82.8 | 0.680 |
| t-PAIC+TM+TAT+PIC+D-D+FDP | 0.904 (0.860-0.949) | 81.5 | 86.6 | 0.681 |
| D-D+FDP | 0.660 (0.569-0.751) | 55.6 | 76.2 | 0.318 |

Abbreviation: GC: Gastric cancer; t-PAIC: Tissue plasminogen activator inhibitor complex; TAT: Thrombin-antithrombin complex; TM: Thrombomodulin; PIC: plasmin-α2-plamininhibitor complex; D-D: D-Dimer; FDP: Fibrinogen degradation product. * P < 0.05 considered statistically significant.
